# Demographic characteristics associated with circadian rest-activity rhythm patterns: a cross-sectional study

**DOI:** 10.1186/s12966-021-01174-z

**Published:** 2021-08-18

**Authors:** Jingen Li, Virend K. Somers, Francisco Lopez-Jimenez, Junrui Di, Naima Covassin

**Affiliations:** 1 Department of Cardiovascular Medicine, Dongzhimen Hospital, Beijing University of Chinese Medicine, Beijing, 100700 China; 2 Department of Cardiovascular Medicine, Mayo Clinic, 200 First Street SW, Rochester, MN 55905 USA; 3 Department of Biostatistics, Johns Hopkins University, Baltimore, MA 21205 USA

**Keywords:** Rest-activity rhythm, Circadian rhythms, Accelerometry, Racial disparities, Sex disparities

## Abstract

**Background:**

Rest-activity rhythm (RAR), a manifestation of circadian rhythms, has been associated with morbidity and mortality risk. However, RAR patterns in the general population and specifically the role of demographic characteristics in RAR pattern have not been comprehensively assessed. Therefore, we aimed to describe RAR patterns among non-institutionalized US adults and age, sex, and race/ethnicity variation using accelerometry data from a nationally representative population.

**Methods:**

This cross-sectional study was conducted using the US National Health and Nutrition Examination Survey (NHANES) 2011–2014. Participants aged ≥20 years who were enrolled in the physical activity monitoring examination and had at least four 24-h periods of valid wrist accelerometer data were included in the present analysis. 24-h RAR metrics were generated using both extended cosinor model (amplitude, mesor, acrophase and pseudo-F statistic) and nonparametric methods (interdaily stability [IS] and intradaily variability [IV]). Multivariable linear regression was used to assess the association between RAR and age, sex, and race/ethnicity.

**Results:**

Eight thousand two hundred participants (mean [SE] age, 49.1 [0.5] years) were included, of whom 52.2% were women and 67.3% Whites. Women had higher RAR amplitude and mesor, and also more robust (pseudo-F statistic), more stable (higher IS) and less fragmented (lower IV) RAR (all *P*_trend_ < 0.001) than men. Compared with younger adults (20–39 years), older adults (≥ 60 years) exhibited reduced RAR amplitude and mesor, but more stable and less fragmented RAR, and also reached their peak activity earlier (advanced acrophase) (all *P*_trend_ < 0.001). Relative to other racial/ethnic groups, Hispanics had the highest amplitude and mesor level, and most stable (highest IS) and least fragmented (lowest IV) RAR pattern (*P*_trend_ < 0.001). Conversely, non-Hispanic blacks had the lowest peak activity level (lowest amplitude) and least stable (lowest IS) RAR pattern (all *P*_trend_ < 0.001).

**Conclusions:**

In the general adult population, RAR patterns vary significantly according to sex, age and race/ethnicity. These results may reflect demographic-dependent differences in intrinsic circadian rhythms and may have important implications for understanding racial, ethnic, sex and other disparities in morbidity and mortality risk.

**Supplementary Information:**

The online version contains supplementary material available at 10.1186/s12966-021-01174-z.

## Background

Circadian rhythms describe the endogenous ~ 24-h oscillations of molecular, physiological and behavioral processes which evolved to synchronize biological functions to the environmental light-dark cycle [1]. Circadian rhythms have been observed in almost all life forms and play a critical role in human health. Disruption of circadian rhythms has been associated with poor health, including increased risk of neurodegenerative diseases [2, 3], metabolic syndrome, diabetes [4, 5], cardiovascular disease [6], and cancer [7].

Rest-activity rhythm (RAR), namely magnitude, timing and regularity of rest-activity patterns, is the most evident manifestation of the circadian rhythm and can be objectively quantified from accelerometry data. In a recent prospective study, accelerometry-derived metrics of RAR including amplitude (strength or magnitude of the rhythm), acrophase (timing of peak activity) and pseudo-F statistic (robustness of the rhythm) independently predicted increased risk of incident diabetes among older men [8]. Another longitudinal analysis of 2930 older men also reported that, compared with participants in the highest quartile of amplitude, mesor (mean activity level) and pseudo-F statistic, those who were in the lowest quartiles had nearly three times greater risk of developing Parkinson disease [9]. Risk of dementia or mild cognitive impairment have also been associated with decreased amplitude or delayed acrophase [10, 11]. Metrics of RAR irregularity such as decreased interdaily stability (IS, day-to-day stability of RAR) and increased intradaily variability (IV, fragmentation of RAR) have been linked with increased risk of cardiometabolic disorders [12], neurodegenerative diseases [3, 13], and mortality [14–16], thus underscoring the critical role of RAR for human health.

Nonetheless, little is known about RAR patterns among general adults and its configuration by sex, age and race/ethnicity. Most of the population-level evidence from accelerometry is limited to standard physical activity levels [17–19]. A small study [20] with 590 adults (80.6% women) assessed RAR patterns and did not observe changes of RAR amplitude with aging, contrary to established knowledge on age-related trajectories in endogenous circadian rhythms [21], possibly because of the small sample size and uneven sex distribution. Therefore, there is need for studies describing RAR composition in the general populations in real-life settings, given that intrinsic circadian rhythms as assessed by core body temperature or melatonin release varied with demographic characteristics in laboratory settings [21–23].

To address the aforementioned gaps, the present study aimed to describe RAR patterns among general adults and to explore variations by sex, age and race/ethnicity using accelerometry data from a large sample from the US National Health and Nutrition Examination Survey (NHANES) 2011–2012 and 2013–2014.

## Methods

This cross-sectional study was conducted and reported following recommendation of the Strengthening the Reporting of Observational Studies in Epidemiology (STROBE) statement [24].

### Data source and population

NHANES provides nationally representative data on nutrition and health of non-institutionalized US civilians obtained from in-person interviews and physical examinations. A stratified, multistage probability sampling method is applied for participant selection [25]. For the present study, we pooled data from participants of the NHANES 2011–2012 and 2013–2014 cycles who underwent the accelerometry-based physical activity monitoring examination (*N* = 14,693). Participants with less than 4 valid-day accelerometry data (*n* = 2152) [26], aged less than 20 years (*n* = 4266), or who were pregnant at screening (*n* = 75) were excluded, leaving a total of 8200 participants for the present analysis. A valid-day of accelerometry data was defined as a day with over 80% wear time during a 24-h period [27], and the prediction of device wear status was made through an open-source algorithm [28]. NHANES protocols were approved by the National Center for Health Statistics Ethics Review Board and all participants provided written informed consent.

### Assessment of rest-activity rhythm

Participants were asked to wear the ActiGraph accelerometer (model GT3X+, Pensacola, FL) on their non-dominant wrist continuously across the 24 h for seven consecutive days. The device was programmed to record acceleration data acquired from the x-, y- or z-axes with 80 Hz sampling frequency. Data quality was reviewed by staff from the National Center for Health Statistics (NCHS) and contractors at Northeastern University in Boston, MA. Acceleration measurements from all three axes were summed for each minute as Monitor-Independent Movement Summary (MIMS) units, a non-proprietary and device-independent universal summary metric [29].

**Fig. 1:**
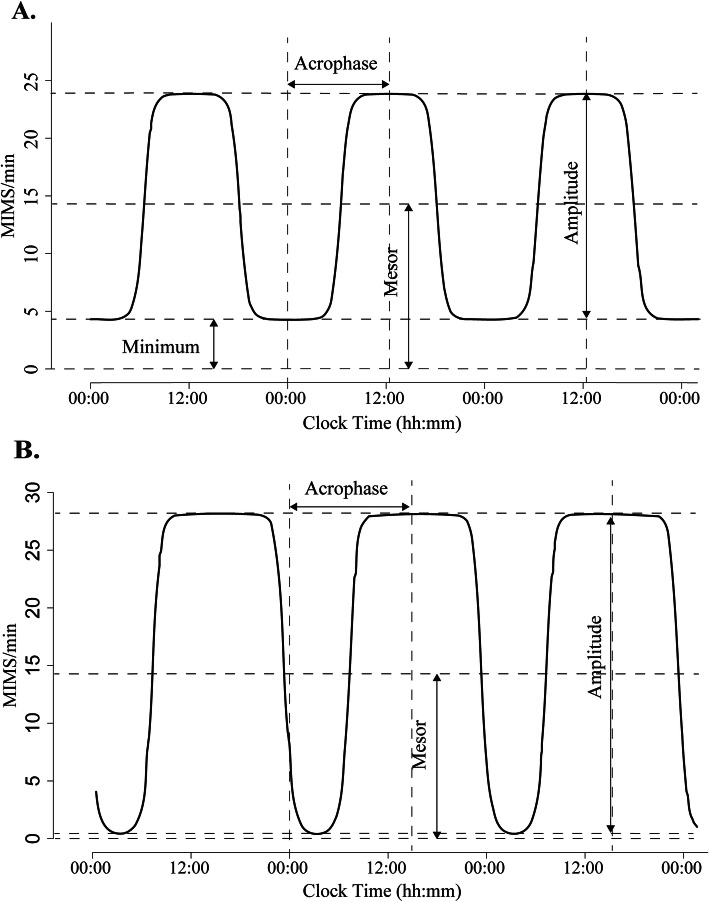
Illustration of rest-activity rhythms of two participants generated using extended cosinor model. For participant **A**: amplitude = 19.54 MIMS/min, mesor = 14.07 MIMS/min, acrophase = 12:19, Pseudo-F statistic = 120.87. For participant **B**: amplitude = 28.06 MIMS/min, mesor = 14.03 MIMS/min, acrophase = 14:55, Pseudo-F statistic = 1058.92

We used two common approaches to quantifying RAR, the extended cosinor model [8, 9, 30] and the nonparametric method [31], excluding the first and last day of accelerometry data due to incomplete 24-h periods (from midnight to midnight). The extended cosinor model applies an antilogistic transformation to the cosinor curve and fits the activity data to a squared wave rather than a cosinor curve [30]. It has been proposed that the extended cosinor model may fit human activity data with higher flexibility since it assumes a shape more similar to a square wave than a cosinor curve [9]. Nonparametric methods do not make distributional or functional assumptions on patterns of rest-activity data, and therefore, may also describe the RAR better than the cosinor model [13]. RAR metrics of the extended cosinor model include the following: 1) amplitude (MIMS/min), measured as peak-to-nadir difference of activity in the fitted curve, represents the peak activity level and is an index of strength of the rhythm; 2) mesor (MIMS/min), is an estimate of the mean activity level based on the fitted curve, and is calculated as minimum value of the function plus 1/2 amplitude; 3) acrophase (clock hours) indicates the timing of peak activity of the fitted curve; 4) pseudo-F statistic, a measure of model goodness of fit, serves as an indicator of robustness of the rhythm. Figure 1 shows pictorial examples of RARs generated by the extended cosinor model using accelerometry data from two participants. The following RAR variables were calculated by nonparametric methods: 1) interdaily stability (IS), a measure of stability of day-to-day RAR, ranges from 0 to 1, with higher values indicating greater stability; 2) intradaily variability (IV), a measure of RAR fragmentation across the 24 h, ranges from 0 to 2 with higher values representing more fragmented rhythms. All RAR metrics were derived from minute-epoch data and detailed definitions of RAR variables are also presented in Additional file 1 Table S1. Missing activity data from non-wear periods were left as zero activity. Acrophase was categorized into three groups in accordance with previous studies [8, 9]: phase advanced (1 SD lower than the mean, i.e., before 12:44), phase delayed (1 SD or greater than the mean, i.e., 16:51 or later) and normal phase (mean ± 1SD, i.e., between 12:44 and < 16:51).

### Population characteristics

Sociodemographic characteristics were obtained by questionnaires during in-home interviews, including age, sex, race/ethnicity, education, employment status, marital status and poverty income ratio. Participants aged 80 years or older were coded as 80 years old in NHANES. Available race/ethnicity categories were ‘Mexican American’, ‘Hispanic’, ‘non-Hispanic white’, ‘non-Hispanic black’ and ‘non-Hispanic Asian’. For the current analysis, we considered respondents who self-reported as ‘Mexican American’ or ‘Hispanic’ as Hispanic. Details of other sociodemographic factors are provided in Additional file 1 Table S2.

### Statistical analysis

To be representative of the US adult population, complex survey design factors including sample weights, clustering, and stratification were taken into account as recommended [32]. Categorical variables are expressed as numbers and weighted percentages and continuous variables are expressed as weighted means and standard errors (SE). RAR metrics are presented as averages for the entire sample as well as according to their quartile distribution and phase categories for acrophase. Differences were tested using the Rao-Scott χ^2^ test for categorical variables and analysis of variance (ANOVA) for continuous variables [33]. Multivariate linear regression models with each of the RAR metrics as dependent variable and each of demographic factors, i.e. age, gender and race/ethnicity as independent variable were fitted, adjusting for education, marital status, employment status and poverty income ratio. Estimated coefficients (β) and 95% confidence interval (CI) for the main predictors are reported. Sociodemographic variables were adjusted for when not tested as exposures. To explore whether the relation between RAR patterns and sex and race/ethnicity changes with age, we also assessed the interaction between sex and age and between race/ethnicity and age using multivariable linear regression. The association between RAR metrics and age stratified by sex and race/ethnicity was also plotted using spline regression, adjusting for the covariates stated above. To rule out potential effects of missing data from non-wear periods, a sensitivity analysis was performed by only including person-days with 100% wear time. Accelerometry data were processed using R (version 4.0.0) with the “ActCR” package [34]. Statistical analyses were performed using SPSS 20.0 (IBM Corp., Armonk, N.Y., USA) and R (version 4.0.0). A two-sided *P* < 0.05 was considered statistically significant.

## Results

### Study population and characteristics

The mean number of valid days of accelerometry data was 6.63 days (range 4 to 7 days) and 90.5% participants had at least 6 days of valid data. Of the total 54,375 person-days, 51,410 (94.5%) person-days had 100% wear time. Of the remaining 2965 (5.5%) person-days with ≤20% of non-wear time, 1399 (47.2%) person-days had 2 h or less of non-wear time (Additional file 1 Fig. S1). Table 1 shows the weighted demographic and RAR characteristics of the study population. Mean age of participants was 49.12 (0.45) years and 52.2% were women. Non-Hispanic whites constituted 67.3% of the population. Demographic characteristics stratified by phases of acrophase and quartiles of amplitude, mesor, pseudo-F statistic, IS and IV are reported in Additional file 1 Tables S3-S5. The population distribution of the RAR metrics is displayed in Additional file 1 Fig. S2.

**Table 1:** Weighted means (SE) of the rest-activity rhythm parameters by population characteristics

Characteristics	No. (%) of participants	Amplitude (MIMS/min)	Mesor (MIMS/min)	Acrophase, (hh:mm)^a^	Pseudo F-statistic	IS	IV
**Total**	8200	13.85 (0.10)	8.10 (0.05)	14:42 (2.7)	240.27 (4.82)	0.366 (0.002)	0.436 (0.001)
**Sex**							
Women	4224 (52.2)	14.02 (0.11)	8.17 (0.05)	14:47 (2.5)	271.62 (6.51)	0.373 (0.002)	0.424 (0.001)
Men	3976 (47.8)	13.67 (0.15)	8.01 (0.08)	14:36 (3.6)	205.97 (4.50)	0.359 (0.002)	0.449 (0.002)
*P*		0.053	0.062	< 0.001	< 0.001	< 0.001	< 0.001
**Age group (years)**							
20–39	2514 (31.8)	15.35 (0.19)	8.97 (0.11)	15:22 (3.9)	215.34 (5.28)	0.358 (0.003)	0.440 (0.002)
40–59	2816 (38.9)	14.29 (0.16)	8.26 (0.08)	14:25 (3.1)	249.15 (6.49)	0.368 (0.003)	0.437 (0.002)
≥ 60	2870 (29.2)	11.63 (0.13)	6.92 (0.08)	14:21 (2.1)	255.58 (8.43)	0.374 (0.002)	0.429 (0.002)
*P*		< 0.001	< 0.001	< 0.001	< 0.001	< 0.001	0.004
**Race/ethnicity**							
Hispanic	1744 (14.3)	16.13 (0.19)	9.34 (0.09)	14:48 (3.9)	219.07 (7.28)	0.386 (0.003)	0.418 (0.003)
NH-White	3367 (67.3)	13.50 (0.14)	7.83 (0.07)	14:35 (3.3)	245.18 (6.65)	0.368 (0.002)	0.437 (0.002)
NH-Black	1918 (11.0)	13.07 (0.18)	8.08 (0.10)	15:00 (2.9)	230.18 (4.31)	0.337 (0.002)	0.446 (0.002)
NH-Asian	933 (4.7)	14.13 (0.27)	8.39 (0.16)	15:05 (4.6)	282.97 (9.93)	0.365 (0.004)	0.433 (0.004)
*P*		< 0.001	< 0.001	< 0.001	< 0.001	< 0.001	< 0.001
**Married or living with a partner**							
Yes	4729 (62.5)	14.03 (0.11)	8.13 (0.06)	14:31 (2.4)	255.20 (6.53)	0.432 (0.002)	0.376 (0.002)
No	3471 (37.5)	13.55 (0.15)	8.04 (0.08)	15:00 (4.2)	215.41 (3.63)	0.442 (0.002)	0.350 (0.002)
*P*		0.007	0.316	< 0.001	< 0.001	< 0.001	< 0.001
**Education**							
Less than high school	1832 (15.9)	14.56 (0.20)	8.52 (0.11)	14:35 (3.0)	214.38 (7.09)	0.431 (0.003)	0.383 (0.003)
High school or equivalent	1809 (21.3)	14.37 (0.20)	8.44 (0.11)	14:33 (4.2)	230.70 (10.17)	0.432 (0.003)	0.375 (0.003)
Some college or AA degree	2486 (32.0)	13.55 (0.19)	7.96 (0.10)	14:54 (5.4)	226.18 (6.78)	0.444 (0.002)	0.359 (0.002)
College graduate or above	2066 (30.8)	13.55 (0.19)	7.96 (0.10)	14:54 (5.4)	226.18 (6.78)	0.444 (0.002)	0.359 (0.002)
*P*		< 0.001	< 0.001	0.18	< 0.001	< 0.001	0.001
**Employed**							
Yes	4324 (59.4)	14.91 (0.16)	8.61 (0.09)	14:31 (3.0)	257.35 (6.45)	0.373 (0.002)	0.432 (0.002)
No	3871 (40.6)	12.29 (0.12)	7.35 (0.06)	14:57 (2.4)	215.19 (4.62)	0.357 (0.002)	0.441 (0.001)
*P*		< 0.001	< 0.001	< 0.001	< 0.001	< 0.001	< 0.001
**Poverty income ratio**							
≥ 1	5815 (84.0)	13.75 (0.12)	8.00 (0.06)	14:37 (2.4)	247.67 (5.89)	0.435 (0.002)	0.367 (0.002)
< 1	1777 (16.0)	14.24 (0.24)	8.50 (0.11)	15:12 (4.8)	198.92 (7.24)	0.443 (0.004)	0.358 (0.004)
*P*		0.091	< 0.001	< 0.001	< 0.001	0.077	0.115

All estimates accounted for complex survey design. Data are presented as number with weighted percentage (%) or weighted means with standard error (SE)

*IS* Interdaily stability, *IV* Intradaily variability, *NH* Non-Hispanic, *SE* Standard error

^a^Clock hours (hh:mm) with standard error (minutes)

### Association between RAR metrics and sex

Compared with men, women had higher amplitude, mesor, pseudo-F statistic and IS values, and lower IV value (Table 1). Consistently, women were more likely to be in the highest quartile of amplitude (53.5% vs. 46.5%), mesor (53.5% vs. 46.5%), pseudo- F statistic (63.0% vs. 37.0%) and IS (57.9% vs. 42.1%) and in the lowest quartile of IV (61.4% vs 38.6%) than men (Additional file 1 Table S3–5). Women were also more likely to be in the normal acrophase than men (54.1% vs 45.9%) (Additional file 1 Table S4). Sex differences in RAR metrics remained evident in multivariable analysis (all *P’*s < 0.001), except for acrophase (*P* = 0.06) (Table 2).

**Table 2:** Coefficients of sex, age and race/ethnicity for rest-activity rhythm metrics from multivariable analysis

RAR metrics	Coefficients	Women vs. Men^a^	Age^b^	Hispanics vs NH-whites^c^	NH-blacks vs NH-whites^c^	NH-Asians vs NH-whites^c^
Amplitude	β (95%CI)	0.82 (0.45, 1.19)	−0.08 (− 0.09, − 0.07)	1.30 (0.84, 1.76)	−0.84 (− 1.27, − 0.41)	0.14 (− 0.43, 0.70)
	Adjusted *P*	< 0.001	< 0.001	< 0.001	< 0.001	0.628
Mesor	β (95%CI)	0.39 (0.20, 0.57)	−0.04 (− 0.05, − 0.04)	0.78 (0.54, 1.02)	0.01 (− 0.20, 0.22)	0.30 (− 0.03, 0.62)
	Adjusted *P*	< 0.001	< 0.001	< 0.001	0.940	0.070
Acrophase	β (95%CI)	0.10 (−0.004, 0.20)	−0.03 (− 0.04, − 0.03)	0.003 (− 0.15, 0.16)	0.14 (− 0.05, 0.34)	0.32 (0.14, 0.50)
	Adjusted *P*	0.055	< 0.001	0.972	0.140	0.001
Pseudo-F statistic	β (95%CI)	71.57 (59.59, 83.56)	1.56 (1.14, 1.97)	−5.85 (−28.50, 16.81)	8.62 (−3.95, 21.18)	39.79 (17.60, 61.98)
	Adjusted *P*	< 0.001	< 0.001	0.603	0.172	0.001
IS	β (95%CI)	0.02 (0.015, 0.025)	0.001 (0.0003, 0.001)	0.015 (0.009, 0.022)	−0.025 (−0.031, −0.018)	− 0.001 (− 0.008, 0.006)
	Adjusted *P*	< 0.001	< 0.001	< 0.001	< 0.001	0.755
IV	β (95%CI)	−0.027 (− 0.031, − 0.023)	−0.0004 (− 0.001, − 0.0003)	−0.020 (− 0.027, − 0.014)	0.005 (− 0.001, 0.011)	−0.004 (− 0.012, 0.003)
	Adjusted *P*	< 0.001	< 0.001	< 0.001	0.088	0.249

*IS* Interdaily stability, *IV* Intradaily variability, *NH* Non-Hispanic, *RAR* Rest-activity rhythm

^a^Adjusted for age, race, education, marital status, employment status, poverty income ratio

^b^ Adjusted for sex, race, education, marital status, employment status, poverty income ratio

^c^Adjusted for sex, age, education, marital status, employment status, poverty income ratio

### Association between RAR metrics and age

In older adults (≥60 years), amplitude, mesor and IV values were lower while pseudo-F statistic and IS values were higher than in both younger adults (20–39 years) and middle-aged adults (40–59 years) (Table 1). Similarly, older adults were more likely to be in the lowest quartiles of amplitude, mesor and IV (Additional file 1 Table S3 and Table S5) than younger and middle-aged adults, but also more likely to be in the highest quartile of pseudo-F statistic and IS (Additional file 1 Table S4–5). Older adults reached their peak activity earlier than younger adults (Acrophase, 14:21 vs 15:22; Table 1) and were more likely to be in advanced phase (36.5% vs 17.4%) (Additional file 1 Table S4). In multivariable linear analysis, age was inversely associated with amplitude, mesor, acrophase and IV, and was positively associated with pseudo-F statistic and IS (Table 2).

### Association between RAR metrics and race/ethnicity

Hispanics had the highest amplitude, mesor, and IS and lowest IV, whereas non-Hispanic blacks had the lowest amplitude and IS and highest IV. Consistently, Hispanics were more likely to be in the highest quartiles of amplitude, mesor, and IS and lowest quartiles of IV, whereas non-Hispanic blacks were more likely to be in the lowest quartile of amplitude, IS and highest quartiles of IV (Additional file 1 Table S3 and Table S5).

Relative to non-Hispanic whites, Hispanics had significantly higher amplitude, mesor, and IS and significantly lower IV independent of covariates (Table 2). Conversely, non-Hispanic blacks had significantly lower amplitude and IS than non-Hispanic whites. Non-Hispanic Asians had a significantly more delayed acrophase than non-Hispanic whites (β, 0.32; *P* = 0.001) (Table 2).

### Interaction between sex, race/ethnicity and age and sensitivity analysis

**Fig. 2:**
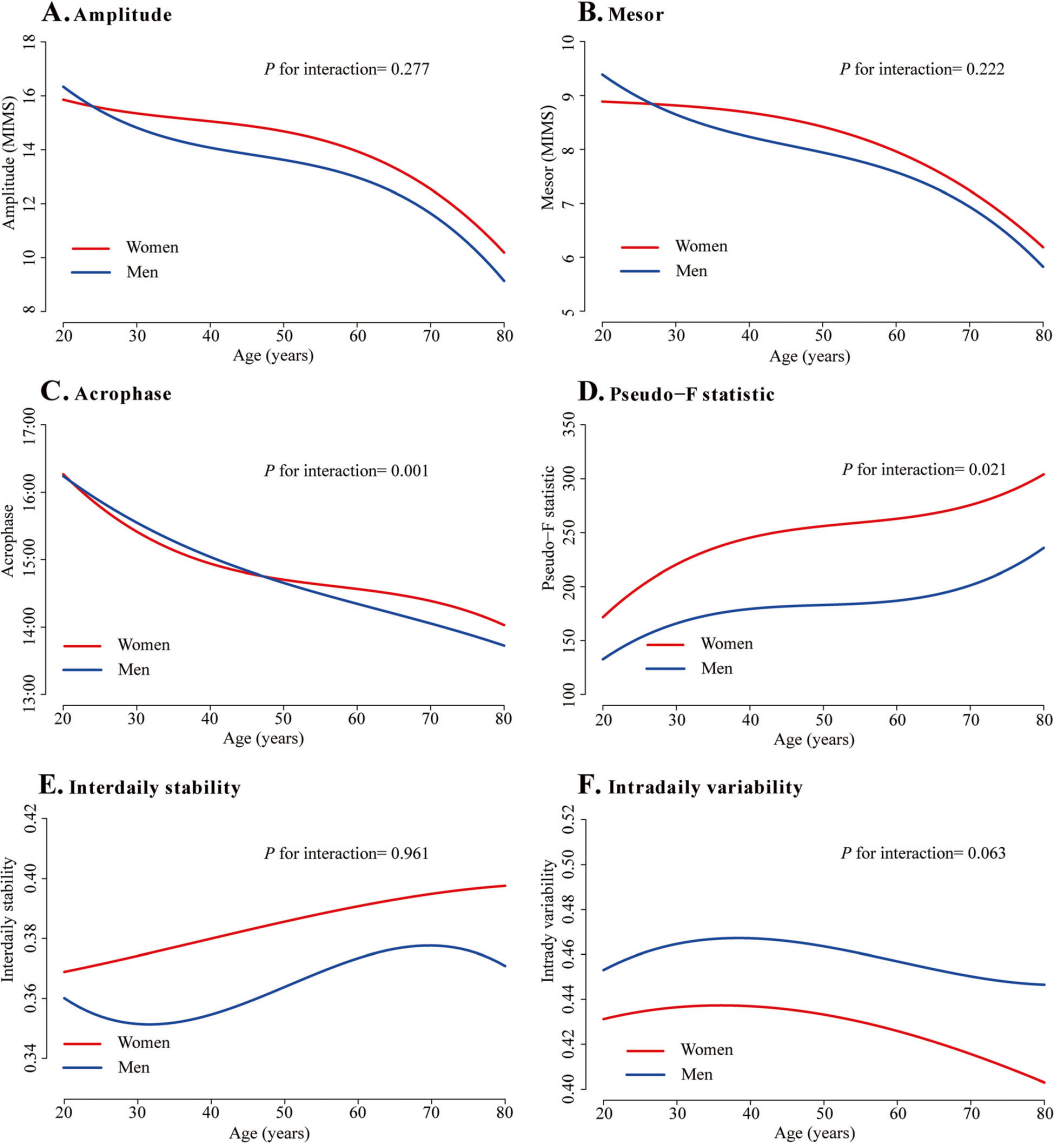
Rest-activity rhythm metrics in men and women across age groups. Graphs were plotted using spline regression adjusting for race, education, marital status, employment status and poverty income ratio

**Fig. 3:**
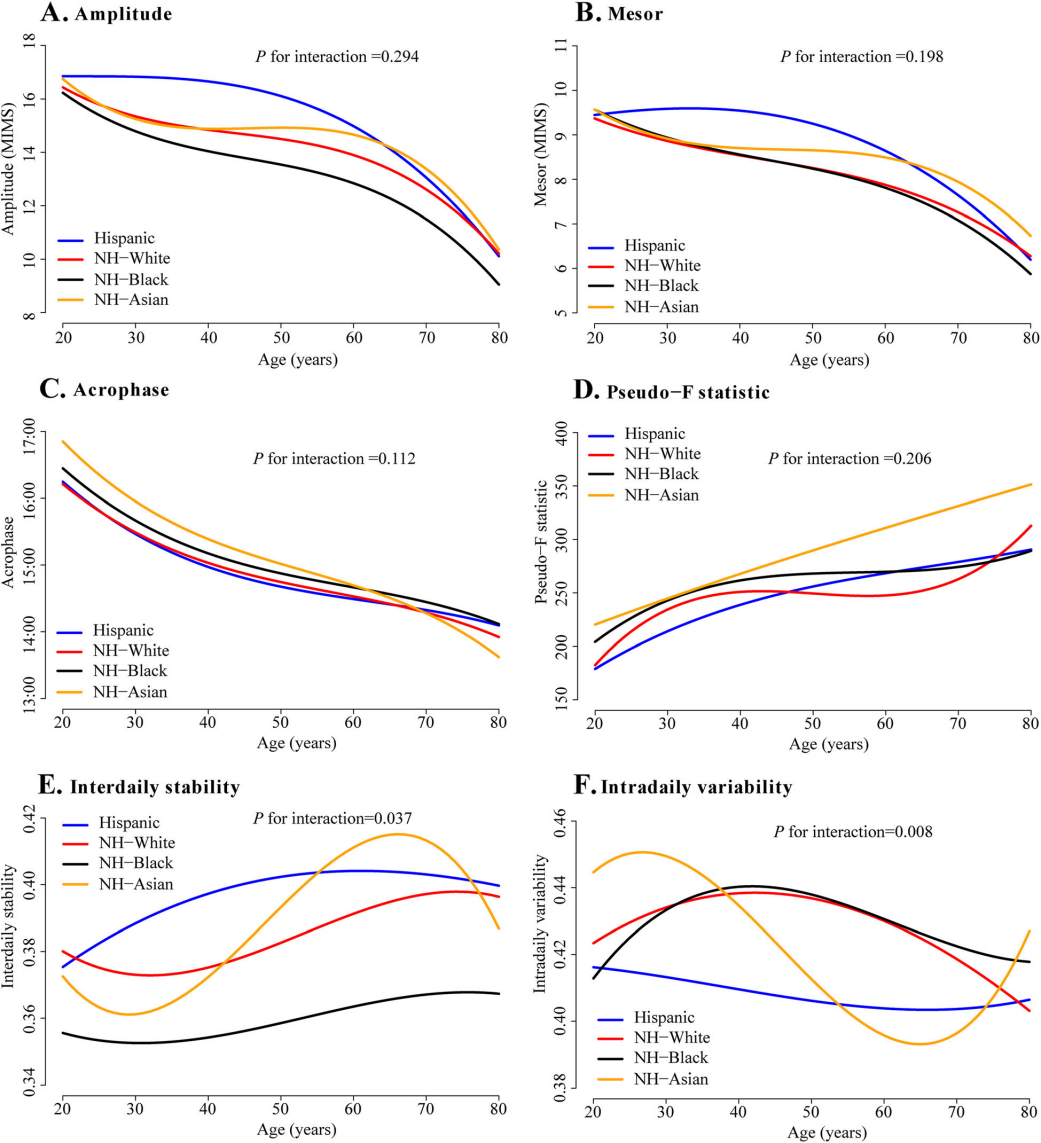
Rest-activity rhythm metrics in Hispanics, NH-whites, NH-blacks and NH-Asians across age groups. Graphs were plotted using spline regression adjusting for sex, education, marital status, employment status and poverty income ratio. NH, non-Hispanic

The interaction analyses showed that acrophase advance with age was more striking in men than women (*P* = 0.001, Fig. 2C, Additional file 1 Table S6) and sex differences in pseudo-F statistic were more evident during middle age (*P* = 0.02, Fig. 2D, Additional file 1 Table S6). There were no significant interactions between sex and age on other RAR metrics. Interactions between race/ethnicity and age were observed for IS (*P* = 0.04) and IV (*P* = 0.01), showing a more pronounced increase in IS and decrease in IV in older non-Hispanic Asians (Fig. 3E-F, Additional file 1 Table S7).

Sensitivity analysis including only person-days with 100% wear time showed similar results compared to the overall sample (Additional file 1 Table S8).

## Discussion

In this large, nationally representative US sample we found that women had higher RAR amplitude and mesor and also had a more stable and less fragmented rest-activity circadian rhythm than men. We also observed lower RAR amplitude and mesor, and more advanced RAR acrophase along with more stable and less fragmented RAR in older adults than in younger adults. RAR also varied among race/ethnicity, with the highest amplitude and mesor levels, and most stable (highest IS) and least fragmented (lowest IV) RAR observed in Hispanics, while the lowest amplitude and most unstable RAR was observed in non-Hispanic blacks. To our knowledge, this is the first study to systematically describe demographic factors associated with RAR patterns in a real-life setting using a large and, importantly, representative sample of US adults.

In line with findings from the UK Biobank and the FinHealth 2017 Survey [18, 19], accelerometry data showed slightly higher amplitude and mesor in women relative to men, indicating higher physical activity as estimated from the fitted curve. However, other reported men having higher or similar accelerometer-measured total physical activity levels compared with women [35, 36], with men being more likely to be sedentary and engaged in moderate-to-vigorous activities and women in light activities. Such discrepancy may be owed to the approach used to determine activity. Because amplitude and mesor are circadian rhythm-adjusted estimates of physical activity based on the fitted curve derived from the extended cosinor model, they may not align with conventionally measured physical activity levels, and especially may not reflect short bursts of vigorous activity. A prior small study showed that women had more stable RAR but showed similar fragmentation levels compared with men, possibly due to the small proportion of male participants (19.4%) and the small sample size (*n* = 590) [20]. Supporting this hypothesis and in line with our results, men were found to have a more fragmented RAR in a study with more balanced sex distribution [13]. The more stable RAR pattern in women could potentially result from the more frequent involvement of regular household activities [37]. When we explored interactions between sex and age, we found that the age-related RAR phase advance was attenuated in women compared to men. This aligns with data from a Spanish population study on chronotypes, showing that, in participants aged > 40 years, women are more evening-oriented [38]. Although underlying mechanisms are unclear, this observation may reflect potential effects of estrogens on the intrinsic circadian rhythm. The slightly shorter circadian period exhibited by women in comparison to men, which results in earlier intrinsic circadian phase [23, 39, 40], has been attributed to their higher level of estrogens. As estrogen levels decrease with aging, their shortening effects on the circadian period may be blunted, therefore leading to less striking phase advance in women than men. Sociocultural factors could also contribute to the observed sex differences in acrophase changes with aging. For instance, prior to retirement years, women may engage in housework and/or childcare activities before going to work in the early morning hours [38].

The association between aging and changes in circadian rhythm has been well established. Briefly, the phase of endogenous circadian rhythms advances and the amplitude dampens with aging [21]. Our findings that mesor and amplitude of RAR decreased while phase advanced in older adults are consistent with these data. Moreover, environmental factors such as reduced work-related physical activity [41] and diminished social interaction [42] may also contribute to decreased amplitude and mesor in older adults. On the other hand, we also unexpectedly found that older adults had more stable and less fragmented RAR than younger adults. This result is seemingly at variance with evidence of decreases in stability and increases in fragmentation relating to age-related neurodegenerative disorders, such as Parkinson’s disease [43] and Alzheimer’s disease [11, 13]. However, the association between circadian rhythms and neurodegenerative disease has been reported to be bidirectional, with neurodegenerative disease also affecting circadian rhythms [1, 3]. Moreover, the present study aimed to assess the association of RAR with age in the general population, which may account for the discrepancy. Accordingly, many studies showed decreased variability in activity in older adults [20, 43–45]. It is plausible that the higher interdaily stability and lower intradaily variability of RAR in older adults may reflect their more rigid day-to-day routine.

Little is known about differences in RAR patterns associated with race/ethnicity [20, 46, 47]. Similar to our findings, a previous study with 590 adults showed that African-Americans and Asians had less stable and weaker accelerometry-derived rest-activity patterns compared to Whites [20]. Mechanisms of the observed racial/ethnic RAR disparities may be complex, resulting from both endogenous and exogenous factors. Genetic differences or biological factors could be potential cause for the observed racial/ethnic variations in our findings [22, 48], and other unmeasured environmental and/or socioeconomic factors, such as work stability, could also be contributors to the observed racial/ethnic pattern of RAR. Although prior laboratory studies [46, 47, 49] reported that, compared with white individuals, African-Americans had slightly shorter intrinsic circadian period (about 13 to 16 min shorter), which often lead to phase advancing [50], we did not find significant differences in acrophases between non-Hispanic blacks versus non-Hispanic whites. Similarly, a recent study also reported no significant difference between whites and blacks on circadian rhythms phase measured by dim light melatonin onset in free-living environment [51]. Potential explanation could be that entrainment of environmental factors may offset the minor circadian rhythms phase difference between the white and black populations. A significantly more delayed phase of RAR in non-Hispanic Asians compared with non-Hispanic whites was observed. Additionally, we noted that the increase in IS and decrease of IV seeing with aging was accentuated in NH-Asians compared to other racial/ethnic groups. Environmental determinants, such as immigration and lifestyle changes, may play a role in this findings, as we found that in this sample the proportion of Asians who lived in US 10 years or longer also increased with age (Additional file 1 Fig. S3). Studies have reported that Asians or Asian immigrants are usually sedentary during their leisure time and longer duration of residence in US has been linked to more leisure time physical activity [52, 53], potentially reflecting the process of settling down into new environment and culture. However, potential mechanisms remain speculative and further studies on racial/ethnic circadian rhythms disparities are needed.

Our findings provide important and novel insights into characteristics of RAR and their potential role on health and disease. First, we report that RAR at least partially reflected the age and sex variation of endogenous circadian rhythms. Supporting our findings, significant correlations between fragmentation of RAR and decreased amplitude of melatonin secretion [54] and between RAR acrophase and cortisol acrophase [55] have been observed. Hence, RAR metrics derived from accelerometry data may serve as complementary marker for assessing endogenous circadian rhythms patterning in epidemiological studies and our data provided a compelling hypothesis-generating foundation for future studies of endogenous circadian variations and their health implications among different populations. Second, the different patterns of RAR we observed based on sex and race/ethnicity may have implications for understanding and potentially addressing disparities in risk of diseases and premature death [56]. Likewise, the higher activity levels and most stable RAR exhibited by Hispanics may at least partially account for the well-known ‘Hispanic paradox’, according to which US Hispanic populations live longer than Whites despite their socioeconomic disadvantages [57, 58]. Our observations that non-Hispanic blacks had the lowest RAR amplitude and interdaily stability are also consistent with the greater risk of premature mortality [57, 59] suffered by African-Americans compared with other ethnic/racial groups. Last, as disruption of RAR has been associated with increased risk of neurodegenerative diseases [3, 13], cardiometabolic diseases [12] and cancer [7], findings from the present study support targeting RAR in preventive and therapeutic strategies to enhance public health.

### Strength and limitation

Strengths of the current study include high device-wearing compliance with 90.5% participants with at least 6 days of valid data, long device wearing periods and use of a large and nationally representative sample, which enabled us to estimate demographic characteristics associated with RAR in free-living environment. By using both an extended cosinor model and non-parametric methods we performed a comprehensive assessment of RAR patterns, describing strength, timing and regularity, which are generalizable to the US population. Our study also has several limitations. First, we acknowledge that accelerometry-derived RAR metrics may be influenced by several environmental and sociodemographic factors in addition to endogenous circadian rhythms. Although we have adjusted for common sociodemographic factors, unmeasured environmental factors could have affected our findings. Second, as this is a cross-sectional study, differences in RAR measures between older and younger individuals that we attributed to age could potentially reflect cohort effects or other confounders. In addition, the observed association between age and RAR metrics may not generalize to the very elderly because individuals aged 80 years or older are top-coded at 80 years in NHANES. Therefore, findings regarding age-dependent differences in RAR should be interpreted cautiously and need further exploration. Third, because accelerometry data from NHANES 2011–2014 are provided as MIMS-units rather than the previously widely used count-units, direct comparisons of RAR metrics such as ‘amplitude’ with previous studies cannot be performed. However, MIMS-units are a non-proprietary and device-independent universal summary metric which have been previously validated to enable comparisons of raw data from different devices (even consumer devices) [29]. Lastly, we did not impute missing data from non-wear periods which might potentially have led to underestimation of RAR metrics. However, missingness was minimal, as 94.5% of person-days had 100% wear time. Furthermore, results of a sensitivity analysis including only person-days with 100% wear time were comparable to those obtained from the entire sample, confirming robustness of our findings.

In conclusion, among the general adult population, RAR patterns vary significantly according to sex, age and race/ethnicity. These results may reflect demographic-dependent differences in intrinsic circadian rhythms and may have important implications for understanding, preventing, and treating disparities in morbidity and mortality risk.

## Supplementary Information

**Additional file 1: Figure S1.** Distribution of amounts of missing time (minutes) among person-days with non-wear time (*n* = 2965 person-days [5.5% of total sample]). **Figure S2.** Population distribution of rest-activity rhythm metrics (*N*=8200). **Figure S3.** Weighted percentage of NH-Asians living in US 10 years or longer across age groups in NHANES 2011–2014. **Table S1.** Definition of rest-activity rhythm metrics. **Table S2.** Coding of sociodemographic factors. **Table S3.** Population characteristics by quartiles of amplitude and mesor. **Table S4.** Population characteristics by phases of acrophase and quartiles of pseudo-F statistic. **Table S5.** Population characteristics by quartiles of interdaily stability and intradaily variability. **Table S6.** Weighted means of rest-activity rhythm metrics in men and women across age groups. **Table S7.** Weighted means of rest-activity rhythm metrics in race/ethnicity categories across age groups. **Table S8.** Adjusted coefficients of the association between rest-activity rhythm metrics and sex, age and race/ethnicity in participants with 100% wear time.

**Additional file 2.** STROBE checklist.

## Data Availability

The datasets used and/or analysed during the current study are available from the corresponding author on reasonable request.

## Abbrevation

AA: Associate of Arts
CI: Confidence interval
NH: Non-Hispanic
NHANES: The US National Health and Nutrition Examination Survey
IS: Interdaily stability
IV: Intradaily variability
MIMS: Monitor-Independent Movement Summary
RAR: Rest-activity rhythm
SD: Standard deviation
SE: Standard error
