# Supplementary material for: Demographic characteristics associated with circadian rest-activity rhythm patterns: a cross-sectional study
Source: Int J Behav Nutr Phys Act. 2021 Aug 18;18:107. doi: 10.1186/s12966-021-01174-z (PMC8371768; doi:10.1186/s12966-021-01174-z)
Supplement: Supplementary file 1 — Additional file 1: Figure S1. Distribution of amounts of missing time (minutes) among person-days with non-wear time (n = 2965 person-days [5.5% of total sample]). Figure S2. Population distribution of rest-activity rhythm metrics (N=8200). Figure S3. Weighted percentage of NH-Asians living in US 10 years or longer across age groups in NHANES 2011–2014. Table S1. Definition of rest-activity rhythm metrics. Table S2. Coding of sociodemographic factors. Table S3. Population characteristics by quartiles of amplitude and mesor. Table S4. Population characteristics by phases of acrophase and quartiles of pseudo-F statistic. Table S5. Population characteristics by quartiles of interdaily stability and intradaily variability. Table S6. Weighted means of rest-activity rhythm metrics in men and women across age groups. Table S7. Weighted means of rest-activity rhythm metrics in race/ethnicity categories across age groups. Table S8. Adjusted coefficients of the association between rest-activity rhythm metrics and sex, age and race/ethnicity in participants with 100% wear time. [file 12966_2021_1174_MOESM1_ESM.docx]

**Supplemental materials**

**Demographic characteristics associated with circadian rest-activity rhythm patterns: A cross-sectional study**

Jingen Li, MD, PhD; Virend K. Somers, MD, PhD; Francisco Lopez-Jimenez, MD, MSc; Junrui Di, PhD; Naima Covassin, PhD*

Correspondence:

Dr. Naima Covassin, Ph.D.

Mayo Clinic College of Medicine

200 First Street SW

Rochester, MN 55905

Phone: 507-255-8897

Email: Covassin.Naima@mayo.edu

**Content**

| **Supplemental figures and tables** | **Page** |
| --- | --- |
| **Figure S1**. Distribution of amounts of missing time (minutes) among person-days with non-wear time (n=2965 person-days [5.5% of total sample]). | 3 |
| **Figure S2**. Population distribution of rest-activity rhythm metrics (N=8200). | 4 |
| **Figure S3**. Weighted percentage of NH-Asians living in US 10 years or longer across age groups in NHANES 2011-2014. | 5 |
| **Table S1**. Definition of rest-activity rhythm metrics. | 6 |
| **Table S2**. Coding of sociodemographic factors. | 7 |
| **Table S3**. Population characteristics by quartiles of amplitude and mesor. | 8-9 |
| **Table S4**. Population characteristics by phases of acrophase and quartiles of pseudo-F statistic. | 10-11 |
| **Table S5**. Population characteristics by quartiles of interdaily stability and intradaily variability. | 12-13 |
| **Table S6**. Weighted means of rest-activity rhythm metrics in men and women across age groups. | 14 |
| **Table S7**. Weighted means of rest-activity rhythm metrics in race/ethnicity categories across age groups. | 15-16 |
| **Table S8.** Adjusted coefficients of the association between rest-activity rhythm metrics and sex, age and race/ethnicity in participants with 100% wear time. | 17 |

**
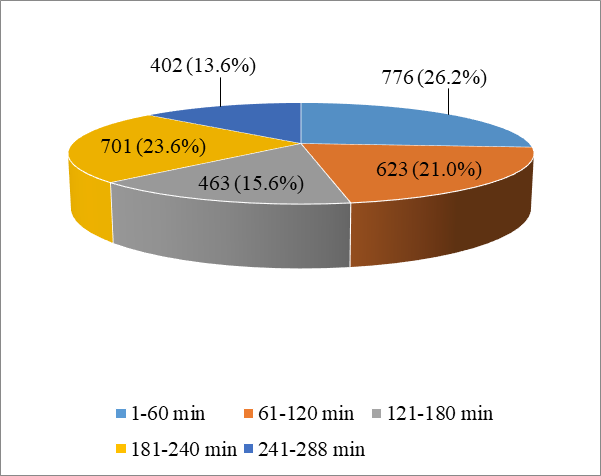
**

**Figure S1. Distribution of amounts of missing time (minutes) among person-days with non-wear time (n=2965 person-days [5.5% of total sample]).** Data are presented as number (%) of person-days.


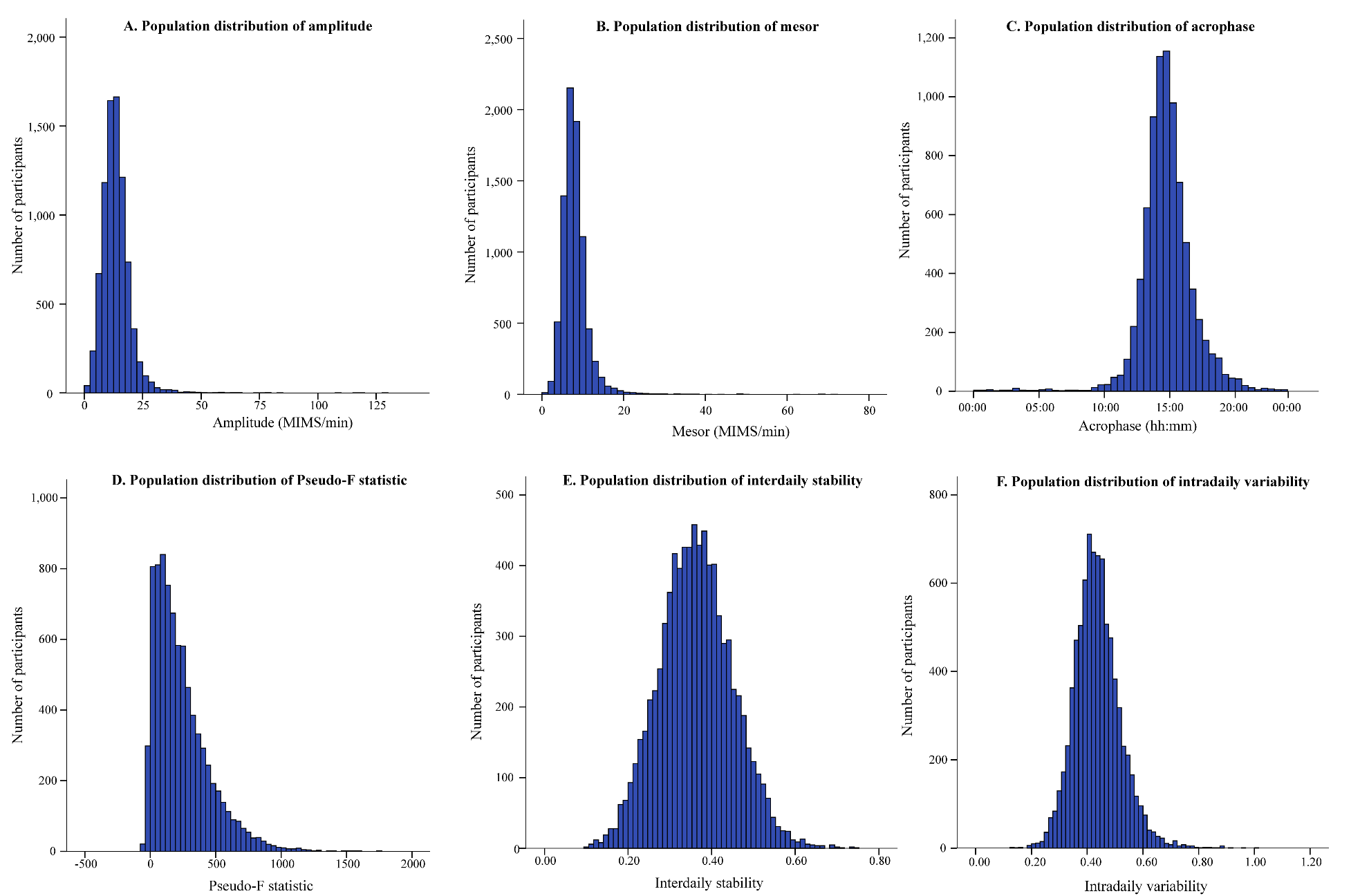


**Figure S2. Population distribution of rest-activity rhythm metrics (N=8200).**


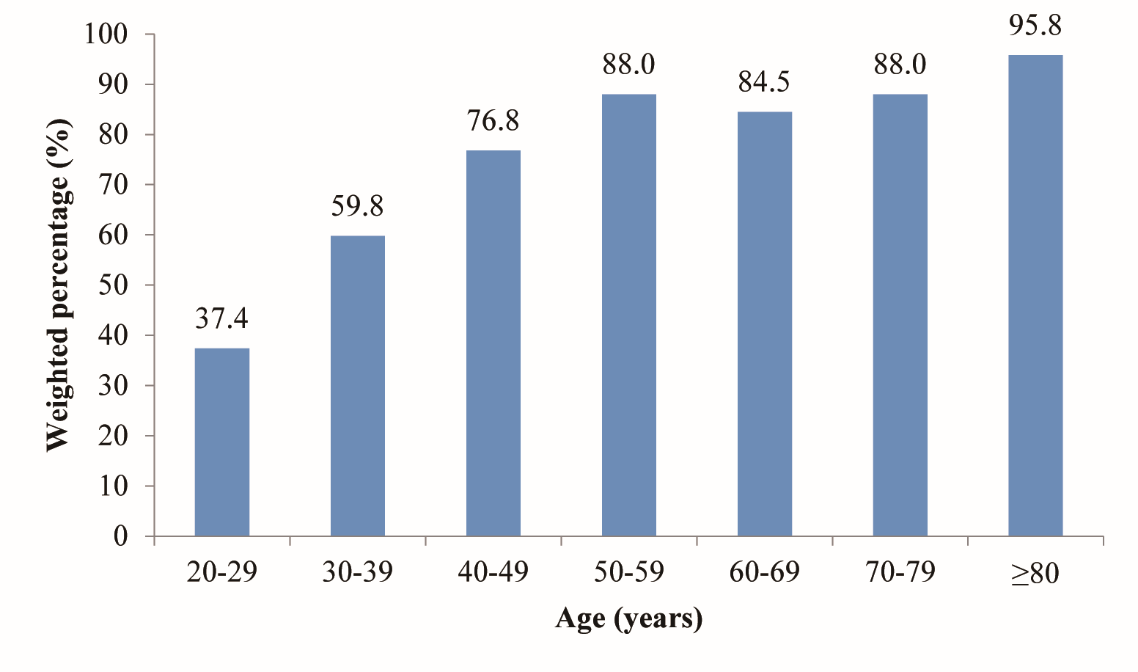


**Figure S3. Weighted percentage of NH-Asians living in US 10 years or longer across age groups in NHANES 2011-2014.** NH, Non-Hispanic.

**Table S1. Definition of rest-activity rhythm metrics.**

| **Variable** | **Definition** |
| --- | --- |
| Amplitude | Measured as peak-to-nadir difference of the fitted curve, represents the peak activity level and is an index of strength of the rhythm. Higher amplitude values indicate higher activity levels. Measured in arbitrary units of activity (MIMS/min) |
| Mesor | The mean activity level of the fitted curve. Calculated as value of the function minimum plus 1/2 amplitude. Measured in arbitrary units of activity (MIMS/min). |
| Acrophase | Clock time reaching peak activity level of the fitted curve. Measured as decimal hours. Categorized into three groups in accordance with previous studies: phase advanced (1 SD less than mean, before 12:44), phase delayed (1 SD or greater than mean, 16:51 or later) and normal phase (mean ± 1SD, between 12:44 and <16:51). |
| Pseudo-F statistic | Measure of model goodness of fit, serves as an indicator of robustness of the rhythm. |
| Interdaily stability | Measure of stability of day-to-day RAR, ranges from 0 to 1, with higher values indicating greater stability. |
| Intradaily variability | Measure of RAR fragmentation across the 24 hours, ranges from 0 to 2, with higher values representing more fragmented rhythms. |

**Table S2. Coding of sociodemographic factors.**

| Education | Categorized into 4 categories: less than high school, high school or equivalent, some college or Associate of Arts (AA) degree, college graduate or above |
| --- | --- |
| Employment status | Determined by the question ‘Which of the following were you doing last week?’. Those who responded ‘working at a job or business’ or ‘with a job or business but not at work’ were regarded as employed, while those who answered ‘looking for a job’ or ‘not working at a job or business’ were considered as unemployed. |
| Marital status | Recoded as a binary variable as follows: married /living with a partner (‘Married’ or ‘living with a partner’) or not (‘Widowed’, ‘divorced’, ‘separated’ or ‘never married’) |
| Poverty income ratio | Coded as a binary variable (< 1 or ≥1) |

**Table S3. Population characteristics by quartiles of amplitude and mesor***

| **Characteristics** | **Amplitude** | | | | ***P* _trend_** | **Mesor** | | | | ***P* _trend_** |
| --- | --- | --- | --- | --- | --- | --- | --- | --- | --- | --- |
|  | **Q1**  **(<9.86)** | **Q2**  **(9.86-<12.96)** | **Q3**  **(12.96-<16.30)** | **Q4**  **(>16.30)** |  | **Q1**  **(<6.19)** | **Q2**  **(6.19-<7.64)** | **Q3**  **(7.64-<9.30)** | **Q4**  **(≥9.30)** |  |
| **Sex, n (%)** | | | | | <0.001 |  |  |  |  | <0.001 |
| Women | 876 (44.9) | 1099 (53.8) | 1167 (55.7) | 1082 (53.5) |  | 893 (45.6) | 1047 (50.6) | 1212 (58.7) | 1072 (53.5) |  |
| Men | 1174 (55.1) | 951 (46.2) | 883 (44.3) | 968 (46.5) |  | 1157 (54.4) | 1003 (49.4) | 838 (41.3) | 978 (46.5) |  |
| **Age, n (%)** | | | | | <0.001 |  |  |  |  | <0.001 |
| 20-39 yrs | 377 (20.3) | 555 (27.5) | 706 (34.6) | 876 (43.0) |  | 353 (18.7) | 575 (27.5) | 702 (35.1) | 884 (45.5) |  |
| 40-59 yrs | 500 (29.5) | 747 (41.9) | 751 (40.9) | 818 (41.9) |  | 518 (31.7) | 720 (40.9) | 774 (42.5) | 804 (39.8) |  |
| ≥60 yrs | 1173 (50.2) | 748 (30.5) | 593 (24.5) | 356 (15.0) |  | 1179 (49.6) | 755 (31.6) | 574 (22.4) | 362 (14.7) |  |
| **Race/ethnicity, n (%)** | | | | | <0.001 |  |  |  |  | <0.001 |
| Hispanic | 260 (7.8) | 338 (10.1) | 458 (14.1) | 688 (24.0) |  | 231 (6.5) | 386 (10.9) | 447 (14.2) | 680 (25.4) |  |
| NH-White | 949 (71.1) | 851 (69.9) | 818 (67.5) | 749 (61.4) |  | 1014 (74.6) | 866 (70.5) | 798 (66.6) | 689 (57.7) |  |
| NH-Black | 586 (13.6) | 531 (12.0) | 463 (10.6) | 338 (8.4) |  | 539 (11.9) | 488 (10.8) | 493 (11.5) | 398 (10.2) |  |
| NH-Asian | 197 (4.5) | 269 (5.3) | 248 (4.7) | 219 (4.1) |  | 212 (4.6) | 248 (4.7) | 247 (4.8) | 226 (4.5) |  |
| **Married or living with a partner, n (%)** | | | | | <0.001 |  |  |  |  | <0.001 |
| Yes | 1034 (55.3) | 1185 (62.6) | 1234 (66.4) | 1276 (64.4) |  | 1077 (56.7) | 1218 (65.6) | 1202 (64.1) | 1232 (62.7) |  |
| No | 1016 (44.7) | 865 (37.4) | 816 (33.6) | 774 (35.6) |  | 973 (43.3) | 832 (34.4) | 848 (35.9) | 818 (37.3) |  |
| **Education, n (%)** | | | | | <0.001 |  |  |  |  | <0.001 |
| Less than high school | 463 (21.4) | 345 (20.3) | 445 (24.4) | 579 (33.9) |  | 437 (16.0) | 370 (11.8) | 453 (15.2) | 572 (21.0) |  |
| High school or equivalent | 463 (21.6) | 391 (21.7) | 419 (24.3) | 536 (32.3) |  | 446 (18.8) | 378 (19.1) | 440 (20.3) | 545 (27.1) |  |
| Some college or AA degree | 608 (22.8) | 642 (26.1) | 667 (27.9) | 569 (23.2) |  | 607 (32.2) | 661 (32.9) | 628 (32.0) | 590 (30.8) |  |
| College graduate or above | 511 (21.9) | 671 (30.7) | 519 (27.2) | 365 (20.2) |  | 555 (33.0) | 640 (36.2) | 529 (32.5) | 342 (21.1) |  |
| **Employed, n (%)** | | | | | <0.001 |  |  |  |  | <0.001 |
| Yes | 641 (38.7) | 1068 (59.6) | 1165 (63.0) | 1450 (73.3) |  | 649 (40.5) | 1099 (61.1) | 1169 (63.2) | 1407 (71.6) |  |
| No | 1408 (61.3) | 982 (40.4) | 883 (37.0) | 598 (26.7) |  | 1400 (59.5) | 950 (38.9) | 880 (36.8) | 641 (28.4) |  |
| **Poverty income ratio, n (%)** | | | | | 0.001 |  |  |  |  | <0.001 |
| ≥1 | 1466 (83.7) | 1525 (86.0) | 1476 (85.7) | 1348 (80.4) |  | 1504 (85.0) | 1547 (87.7) | 1434 (84.1) | 1330 (78.9) |  |
| <1 | 436 (16.3) | 404 (14.0) | 428 (14.3) | 509 (19.6) |  | 413 (15.0) | 378 (12.3) | 457 (15.9) | 529 (21.1) |  |

*All estimates accounted for complex survey design. Data are presented as number with weighted percentage (%).

AA, Associate of Arts degree.

**Table S4. Population characteristics by phases of acrophase and quartiles of pseudo-F statistic ***

| **Characteristics** | **Acrophase^#^** | | | ***P* _trend_** | **Pseudo-F statistic** | | | | ***P* _trend_** |
| --- | --- | --- | --- | --- | --- | --- | --- | --- | --- |
|  | **Advanced (<12:44)**  **n=722** | **Normal**  **(12:44 to <16:51)**  **n=6515** | **Delayed (≥16:51)**  **n=963** |  | **Q1**  **(<81.26)** | **Q2 (81.26-<186.07)** | **Q3 (186.07-<339.40)** | **Q4 (≥339.40)** |  |
| **Sex, n (%)** | | | | <0.001 |  |  |  |  | <0.001 |
| Women | 261 (37.9) | 3508 (54.1) | 455 (48.8) |  | 920 (44.6) | 961 (47.5) | 1065 (53.5) | 1278 (63.0) |  |
| Men | 461 (62.1) | 3007 (45.9) | 508 (51.2) |  | 1130 (55.4) | 1089 (52.5) | 985 (46.5) | 772 (37.0) |  |
| **Age, n (%)** | | | | <0.001 |  |  |  |  | <0.001 |
| 20-39 yrs | 118 (17.4) | 1848 (29.7) | 548 (60.2) |  | 749 (9.4) | 635 (7.9) | 591 (7.2) | 539 (7.3) |  |
| 40-59 yrs | 276 (46.1) | 2287 (39.8) | 253 (26.1) |  | 680 (36.4) | 717 (39.7) | 681 (38.7) | 738 (40.8) |  |
| ≥60 yrs | 328 (36.5) | 2380 (30.5) | 162 (13.8) |  | 621 (24.9) | 698 (28.7) | 778 (32.7) | 773 (30.5) |  |
| **Race/ethnicity, n (%)** | | | | <0.001 |  |  |  |  | <0.001 |
| Hispanic | 196 (16.9) | 1356 (13.5) | 192 (18.4) |  | 480 (16.8) | 442 (14.9) | 412 (13.2) | 410 (12.4) |  |
| NH-White | 280 (66.6) | 2757 (68.9) | 330 (54.9) |  | 850 (65.6) | 836 (66.0) | 850 (68.3) | 831 (69.2) |  |
| NH-Black | 173 (11.4) | 1462 (10.3) | 283 (17.1) |  | 479 (11.5) | 490 (11.0) | 492 (11.3) | 457 (10.5) |  |
| NH-Asian | 63 (3.6) | 748 (4.5) | 122 (6.3) |  | 177 (3.6) | 212 (4.2) | 241 (4.8) | 303 (5.9) |  |
| **Married or living with a partner, n (%)** | | | | <0.001 |  |  |  |  | <0.001 |
| Yes | 448 (65.8) | 3879 (64.2) | 402 (46.0) |  | 1045 (55.0) | 1169 (61.4) | 1197 (63.3) | 1318 (69.9) |  |
| No | 274 (34.2) | 2636 (35.8) | 561 (54.0) |  | 1005 (11.0) | 881 (9.7) | 853 (9.3) | 732 (7.6) |  |
| **Education, n (%)** | | | | <0.001 |  |  |  |  | <0.001 |
| Less than high school | 233 (12.5) | 1412 (77.1) | 187 (10.4) |  | 517 (4.5) | 439 (4.2) | 466 (4.0) | 410 (3.2) |  |
| High school or equivalent | 197 (11.2) | 1367 (77.2) | 245 (11.6) |  | 501 (25.3) | 447 (20.7) | 439 (19.5) | 422 (19.8) |  |
| Some college or AA degree | 183 (6.9) | 1942 (80.1) | 361 (12.9) |  | 660 (34.3) | 685 (33.9) | 581 (30.7) | 560 (29.2) |  |
| College graduate or above | 109 (5.1) | 1789 (88.6) | 168 (6.4) |  | 370 (21.8) | 478 (28.7) | 563 (33.9) | 655 (38.5) |  |
| **Employed, n (%)** | | | | <0.001 |  |  |  |  | <0.001 |
| Yes | 462 (70.4) | 3405 (59.4) | 457 (50.8) |  | 1045 (13.5) | 1008 (14.0) | 1059 (14.9) | 1212 (17.1) |  |
| No | 260 (29.6) | 3107(40.6) | 504 (49.2) |  | 1003 (44.7) | 1041 (44.1) | 990 (41.2) | 837 (32.5) |  |
| **Poverty income ratio, n (%)** | | | | <0.001 |  |  |  |  | <0.001 |
| ≥1 | 507 (84.6) | 4709 (85.5) | 599 (71.9) |  | 1362 (78.5) | 1437 (83.8) | 1483 (84.8) | 1533 (88.7) |  |
| <1 | 150(15.4) | 1323 (14.5) | 304 (28.1) |  | 537 (21.5) | 452 (16.2) | 434 (15.2) | 354 (11.3) |  |

*All estimates accounted for complex survey design. Data arere presented as number with weighted percentage (%).

^#^Acrophase was categorized as advanced (mean-1 standard deviation [SD]), normal (mean +/-1 SD), and delayed phase (mean+1 SD). AA, Associate of Arts degree.

| **Table S5. Population characteristics by quartiles of interdaily stability and intradaily variability*** | | | | | | | | | | |
| --- | --- | --- | --- | --- | --- | --- | --- | --- | --- | --- |
| **Characteristics** | **Interdaily stability** | | | |  | **Intradaily variability** | | | |  |
|  | **Q1**  **(<0.30)** | **Q2**  **(0.30-<0.36)** | **Q3**  **(0.36-<0.42)** | **Q4**  **(≥0.42)** | ***P* _trend_** | **Q1**  **(<0.38)** | **Q2**  **(0.38-<0.43)** | **Q3**  **(0.43-<0.48)** | **Q4**  **(≥0.48)** | ***P* _trend_** |
| **Sex, n (%)** | | | | | <0.001 |  |  |  |  | <0.001 |
| Women | 913 (45.5) | 1039 (50.7) | 1122 (53.9) | 1150 (57.9) |  | 1263 (61.4) | 1173 (58.4) | 996 (48.9) | 792 (39.8) |  |
| Men | 1137 (54.5) | 1011 (49.3) | 928 (46.1) | 900 (42.1) |  | 787 (38.6) | 877 (41.6) | 1054 (51.1) | 1258 (60.2) |  |
| **Age, n (%)** | | | | | 0.001 |  |  |  |  | 0.002 |
| 20-39 yrs | 703 (37.1) | 658 (32.5) | 610 (31.1) | 543 (27.4) |  | 533 (26.9) | 629 (32.4) | 688 (34.5) | 664 (33.5) |  |
| 40-59 yrs | 664 (36.7) | 710 (40.6) | 697 (38.3) | 745 (39.8) |  | 710 (38.6) | 711 (39.4) | 690 (37.5) | 705 (40.3) |  |
| ≥60 yrs | 683 (26.2) | 682 (26.8) | 743 (30.6) | 762 (32.8) |  | 807 (34.5) | 710 (28.2) | 672 (28.0) | 681 (26.2) |  |
| **Race/ethnicity, n (%)** | | | | | <0.001 |  |  |  |  | <0.001 |
| Hispanic | 313 (2.7) | 370 (3.0) | 427 (3.4) | 634 (5.2) |  | 583 (18.9) | 460 (14.4) | 381 (12.5) | 320 (11.3) |  |
| NH-White | 772 (62.0) | 855 (68.1) | 896 (70.7) | 844 (67.6) |  | 789 (65.6) | 863 (68.8) | 845 (67.9) | 870 (66.9) |  |
| NH-Black | 664 (16.7) | 537 (12.7) | 439 (9.7) | 278 (6.0) |  | 377 (8.6) | 468 (10.6) | 520 (12.2) | 553 (13.0) |  |
| NH-Asian | 221 (5.0) | 229 (4.7) | 232 (4.4) | 251 (4.6) |  | 265 (5.1) | 201 (4.0) | 242 (4.7) | 225 (4.8) |  |
| **Married or living with a partner, n (%)** | | | | | <0.001 |  |  |  |  | 0.001 |
| Yes | 932 (50.0) | 1180 (62.4) | 1246 (65.1) | 1371 (70.5) |  | 797 (34.8) | 839 (35.5) | 874 (38.1) | 961 (41.9) |  |
| No | 1118 (50.0) | 870 (37.6) | 804 (34.9) | 679 (29.5) |  | 1253 (65.2) | 1211 (64.5) | 1176 (61.9) | 1089 (58.1) |  |
| **Education, n (%)** | | | | | <0.001 |  |  |  |  | <0.001 |
| Less than high school | 386 (13.9) | 396 (13.0) | 438 (15.3) | 612 (21.2) |  | 597 (20.4) | 436 (14.6) | 365 (12.5) | 434 (16.2) |  |
| High school or equivalent | 427 (18.2) | 419 (20.7) | 446 (20.6) | 517 (25.3) |  | 477 (23.6) | 453 (21.3) | 451 (20.0) | 428 (20.3) |  |
| Some college or AA degree | 703 (36.9) | 642 (32.6) | 606 (30.8) | 535 (28.3) |  | 493 (26.1) | 633 (31.8) | 668 (34.1) | 692 (36.1) |  |
| College graduate or above | 532 (31.1) | 590 (33.7) | 559 (33.3) | 385 (25.2) |  | 483 (30.0) | 525 (32.3) | 565 (33.4) | 493 (27.4) |  |
| **Employed, n (%)** | | | | | <0.001 |  |  |  |  | 0.002 |
| Yes | 912 (52.0) | 1055 (59.0) | 1127 (62.4) | 1230 (63.2) |  | 1113 (57.6) | 1125 (62.6) | 1137 (62.3) | 949 (55.0) |  |
| No | 1138 (48.0) | 992 (41.0) | 922 (37.6) | 819 (36.8) |  | 936 (42.4) | 924 (37.4) | 910 (37.7) | 1101 (45.0) |  |
| **Poverty income ratio, n (%)** | | | | | 0.021 |  |  |  |  | 0.069 |
| ≥1 | 1434 (81.1) | 1488 (84.4) | 1483 (85.9) | 1410 (84.2) |  | 1410 (83.9) | 1479 (86.3) | 1496 (83.7) | 1430 (82.1) |  |
| <1 | 487 (18.9) | 433 (15.6) | 419 (14.1) | 438 (15.8) |  | 434 (16.1) | 413 (13.7) | 438 (16.3) | 492 (17.9) |  |

*All estimates accounted for complex survey design. Data are presented as number with weighted percentage (%).

AA, Associate of Arts degree.

**Table S6. Weighted means of rest-activity rhythm parameters in men and women across age groups.**

| **RAR Metrics** |  | **Age group (years)** | | | | | | ***P* for interaction** |
| --- | --- | --- | --- | --- | --- | --- | --- | --- |
|  |  | **20-29** | **30-39** | **40-49** | **50-59** | **60-69** | **≥70** |  |
| Amplitude  (MIMS-units/min) | Women | 15.48 | 15.33 | 14.71 | 14.61 | 13.32 | 10.36 | 0.277 |
|  | Men | 15.64 | 14.97 | 14.37 | 13.42 | 12.52 | 9.86 |  |
|  | *P* _trend_ | 0.798 | 0.133 | 0.160 | 0.008 | 0.001 | <0.001 |  |
| Mesor (MIMS-units/min) | Women | 8.96 | 8.99 | 8.47 | 8.39 | 7.78 | 6.35 | 0.222 |
|  | Men | 9.14 | 8.78 | 8.33 | 7.84 | 7.19 | 6.17 |  |
|  | *P* _trend_ | 0.990 | 0.219 | 0.343 | 0.029 | <0.001 | <0.001 |  |
| Acrophase  (hh:mm) | Women | 15:50 | 14:57 | 14:37 | 14:31 | 14:34 | 14:27 | 0.001 |
|  | Men | 15:40 | 15:02 | 14:26 | 14:06 | 14:12 | 14:04 |  |
|  | *P* _trend_ | 0.642 | 0.069 | 0.794 | 0.019 | 0.056 | <0.001 |  |
| Pseudo-F statistic | Women | 195.50 | 280.20 | 283.91 | 285.92 | 292.06 | 279.76 | 0.021 |
|  | Men | 164.93 | 218.53 | 217.72 | 202.13 | 206.27 | 230.96 |  |
|  | *P* _trend_ | 0.001 | <0.001 | <0.001 | <0.001 | <0.001 | <0.001 |  |
| IS | Women | 0.355 | 0.374 | 0.372 | 0.379 | 0.384 | 0.373 | 0.961 |
|  | Men | 0.346 | 0.355 | 0.358 | 0.361 | 0.373 | 0.362 |  |
|  | *P* _trend_ | 0.038 | <0.001 | <0.001 | <0.001 | 0.008 | <0.001 |  |
| IV | Women | 0.434 | 0.424 | 0.429 | 0.422 | 0.419 | 0.414 | 0.063 |
|  | Men | 0.453 | 0.448 | 0.451 | 0.450 | 0.444 | 0.446 |  |
|  | *P* _trend_ | 0.001 | <0.001 | <0.001 | <0.001 | <0.001 | 0.003 |  |

IS, interdaily stability; IV, intradaily variability; NH, non-Hispanic; RAR, rest-activity rhythm.

**Table S7. Weighted means of rest-activity rhythm metrics in race/ethnicity categories across age groups.**

| **RAR metrics** | **Age group (years)** | **20-29** | **30-39** | **40-49** | **50-59** | **60-69** | **≥70** | ***P* for interaction** |
| --- | --- | --- | --- | --- | --- | --- | --- | --- |
| Amplitude (MIMS-units/min) | Hispanic | 16.53 | 17.24 | 17.08 | 15.43 | 14.26 | 10.73 | 0.294 |
|  | NH-White | 15.31 | 14.78 | 14.22 | 14.04 | 12.90 | 10.24 |  |
|  | NH-Black | 15.12 | 13.77 | 13.55 | 13.03 | 11.83 | 8.90 |  |
|  | NH-Asian | 15.03 | 15.03 | 13.83 | 14.45 | 14.02 | 10.59 |  |
|  | *P* _trend_ | 0.339 | 0.006 | <0.001 | 0.010 | <0.001 | 0.001 |  |
| Mesor (MIMS-units/min) | Hispanic | 9.53 | 9.76 | 9.95 | 9.04 | 8.31 | 6.73 | 0.198 |
|  | NH-White | 8.85 | 8.70 | 8.08 | 8.00 | 7.40 | 6.27 |  |
|  | NH-Black | 9.16 | 8.39 | 8.32 | 8.07 | 7.42 | 5.96 |  |
|  | NH-Asian | 8.88 | 8.92 | 8.19 | 8.44 | 8.23 | 6.78 |  |
|  | *P* _trend_ | 0.881 | 0.074 | <0.001 | 0.047 | <0.001 | 0.051 |  |
| Acrophase  (hh:mm) | Hispanic | 15:36 | 14:47 | 14:34 | 14:11 | 14:20 | 14:27 | 0.112 |
|  | NH-White | 15:39 | 14:56 | 14:28 | 14:17 | 14:22 | 14:15 |  |
|  | NH-Black | 16:08 | 15:17 | 14:40 | 14:35 | 14:38 | 14:32 |  |
|  | NH-Asian | 16:18 | 15:28 | 14:49 | 14:36 | 14:34 | 14:00 |  |
|  | *P* _trend_ | 0.232 | 0.020 | 0.068 | 0.484 | 0.457 | 0.023 |  |
| Pseudo-F  statistic | Hispanic | 172.30 | 206.93 | 256.85 | 229.30 | 252.36 | 246.77 | 0.206 |
|  | NH-White | 180.26 | 267.44 | 249.46 | 250.12 | 250.49 | 258.89 |  |
|  | NH-Black | 187.58 | 226.20 | 252.43 | 235.94 | 240.54 | 246.16 |  |
|  | NH-Asian | 200.10 | 282.74 | 301.63 | 266.08 | 356.88 | 329.30 |  |
|  | *P* _trend_ | 0.244 | 0.532 | 0.152 | 0.927 | 0.008 | 0.033 |  |
| IS | Hispanic | 0.362 | 0.393 | 0.404 | 0.386 | 0.394 | 0.379 | 0.037 |
|  | NH-White | 0.354 | 0.364 | 0.361 | 0.373 | 0.382 | 0.371 |  |
|  | NH-Black | 0.330 | 0.330 | 0.338 | 0.345 | 0.341 | 0.338 |  |
|  | NH-Asian | 0.334 | 0.351 | 0.364 | 0.388 | 0.400 | 0.373 |  |
|  | *P* _trend_ | 0.003 | <0.001 | <0.001 | <0.001 | <0.001 | <0.001 |  |
| IV | Hispanic | 0.431 | 0.414 | 0.411 | 0.411 | 0.417 | 0.427 | 0.008 |
|  | NH-White | 0.445 | 0.439 | 0.446 | 0.437 | 0.432 | 0.425 |  |
|  | NH-Black | 0.441 | 0.452 | 0.450 | 0.445 | 0.443 | 0.444 |  |
|  | NH-Asian | 0.468 | 0.444 | 0.429 | 0.413 | 0.404 | 0.428 |  |
|  | *P* _trend_ | <0.001 | 0.001 | 0.005 | 0.009 | <0.001 | 0.154 |  |

IS, interdaily stability; IV, intradaily variability; NH, non-Hispanic; RAR, rest-activity rhythm.

**Table S8. Adjusted coefficients of the association between rest-activity rhythm metrics and sex, age and race/ethnicity in participants with 100% wear time.**

| **RAR metrics** | **Coefficients** | **Women vs. Men^*^** | **Age^#^** | **Hispanics vs NH-whites^†^** | **NH-blacks vs NH-whites^†^** | **NH-Asians vs NH-whites^†^** |
| --- | --- | --- | --- | --- | --- | --- |
| Amplitude | β (95%CI) | 0.72 (0. 35, 1.09) | -0.08 (-0.09, -0.07) | 1.24 (0.79, 1.69) | -0.96 (-1.37, -0.55) | 0.11 (-0.54, 0.75) |
|  | Adjusted P | <0.001 | <0.001 | <0.001 | <0.001 | 0.735 |
| Mesor | β (95%CI) | 0.31 (0.14, 0.49) | -0.04 (-0.05, -0.04) | 0.74 (0.48, 0.99) | -0.04 (-0.26, 00.18) | 0.28 (-0.10, 0.65) |
|  | Adjusted P | <0.001 | <0.001 | <0.001 | 0.715 | 0.139 |
| Acrophase | β (95%CI) | 0.14 (0.03, 0.25) | -0.03 (-0.04, -0.03) | 0.015 (-0.16, 0.19) | 0.15 (-0.05, 0.36) | 0.34 (0.16, 0.52) |
|  | Adjusted P | 0.014 | <0.001 | 0.867 | 0.141 | <0.001 |
| Pseudo-F statistic | β (95%CI) | 64.91 (52.28, 77.55) | 1.51 (1.12, 1.90) | -50.425 (-21.21, 22.05) | 12.36 (0.064, 24.658) | 35.31(14.15, 56.48) |
|  | Adjusted P | <0.001 | <0.001 | 0.968 | 0.049 | 0.002 |
| IS | β (95%CI) | 0.02 (0.016, 0.030) | 0.0003  (0.00008, 0.001) | 0.010 (0.003, 0.018) | -0.027 (-0.034, -0.019) | -0.002 (-0.008, 0.011) |
|  | Adjusted P | <0.001 | <0.0010.008 | 0.008 | <0.001 | 0.755 |
| IV | β (95%CI) | -0.027  (-0.031, -0.023) | -0.0004  (-0.001, -0.0003) | -0.021  (-0.029, -0.014) | 0.004  (-0.002, 0.010) | -0.003  (-0.011, 0.005) |
|  | Adjusted P | <0.001 | <0.001 | <0.001 | 0.156 | 0.492 |

*Adjusted for age, race, education, marital status, employment status, poverty income ratio.

# Adjusted for sex, race, education, marital status, employment status, poverty income ratio.

†Adjusted for sex, age, education, marital status, employment status, poverty income ratio.

IS, interdaily stability; IV, intradaily variability; NH, non-Hispanic; RAR, rest-activity rhythm.
